# Coexistence and prevalence of obstetric interventions: an analysis based on the grade of membership

**DOI:** 10.1186/s12884-021-04092-x

**Published:** 2021-09-09

**Authors:** Karina Cristina Rouwe de Souza, Thales Philipe Rodrigues da Silva, Ana Kelve de Castro Damasceno, Bruna Figueiredo Manzo, Kleyde Ventura de Souza, Maria Margarida Leitão Filipe, Fernanda Penido Matozinhos

**Affiliations:** 1 Graduate Nursing Program, Nursing School, Universidade Federal de Minas Gerais, Belo Horizonte City, Minas Gerais State Brazil; 2 Health Sciences, Child and Adolescent Health, Medical School, Universidade Federal de Minas Gerais, Belo Horizonte City, Minas Gerais State Brazil; 3 Universidade Federal do Ceará, Ceará State Fortaleza City, Brazil; 4 Maternal and Child Nursing and Public Health Department, Nursing School, Universidade Federal de Minas Gerais, Belo Horizonte City, Minas Gerais State Brazil; 5 Nursing - Matosinhos Health Unit, Matosinhos , Portugal

**Keywords:** Obstetric Nursing, Labor, Obstetrics, Parturition, Humanized Delivery, Birth

## Abstract

**Background:**

Obstetric interventions performed during delivery do not reflect improvements in obstetric care. Several practices routinely performed during childbirth, without any scientific evidence or basis - such as Kristeller maneuver, routine episiotomy, and movement or feeding restriction - reflect a disrespectful assistance reality that, unfortunately, remains in place in Brazil. The aims of the current study are to assess the coexistence and prevalence of obstetric interventions in maternity hospitals in Belo Horizonte City, based on the Grade of Membership (GoM) method, as well as to investigate sociodemographic and obstetric factors associated with coexistence profiles generated by it.

**Methods:**

Observational study, based on a cross-sectional design, carried out with data deriving from the study “Nascer em Belo Horizonte: Inquérito sobre o Parto e Nascimento” (Born in Belo Horizonte: Survey on Childbirth and Birth). The herein investigated interventions comprised practices that are clearly useful and should be encouraged; practices that are clearly harmful or ineffective and should be eliminated; and practices that are inappropriately used, in contrast to the ones recommended by the World Health Organization. The analyzed interventions comprised: providing food to parturient women, allowing them to have freedom to move, use of partogram, adopting non-pharmacological methods for pain relief, enema, perineal shaving, lying patients down for delivery, Kristeller maneuver, amniotomy, oxytocin infusion, analgesia and episiotomy. The current study has used GoM to identify the coexistence of the adopted obstetric interventions. Variables such as age, schooling, skin color, primigravida, place-of–delivery financing, number of prenatal consultations, gestational age at delivery, presence of obstetric nurse at delivery time, paid work and presence of companion during delivery were taken into consideration at the time to build patients’ profile.

**Results:**

Results have highlighted two antagonistic obstetric profiles, namely: profile 1 comprised parturient women who were offered diet, freedom to move, use of partogram, using non-pharmacological methods for pain relief, giving birth in lying position, patients who were not subjected to Kristeller maneuver, episiotomy or amniotomy, women did not receive oxytocin infusion, and analgesia using. Profile 2, in its turn, comprised parturient women who were not offered diet, who were not allowed to have freedom to move, as well as who did not use the partograph or who were subjected to non-pharmacological methods for pain relief. They were subjected to enema, perineal shaving, Kristeller maneuver, amniotomy and oxytocin infusion. In addition, they underwent analgesia and episiotomy. This outcome emphasizes the persistence of an obstetric care model that is not based on scientific evidence. Based on the analysis of factors that influenced the coexistence of obstetric interventions, the presence of obstetric nurses in the healthcare practice has reduced the likelihood of parturient women to belong to profile 2. In addition, childbirth events that took place in public institutions have reduced the likelihood of parturient women to belong to profile 2.

**Conclusion(s):**

Based on the analysis of factors that influenced the coexistence of obstetric interventions, financing the hospital for childbirth has increased the likelihood of parturient women to belong to profile 2. However, the likelihood of parturient women to belong to profile 2 has decreased when hospitals had an active obstetric nurse at the delivery room. The current study has contributed to discussions about obstetric interventions, as well as to improve childbirth assistance models. In addition, it has emphasized the need of developing strategies focused on adherence to, and implementation of, assistance models based on scientific evidence.

## Background

Changes observed in the delivery scenario since its institutionalization had impact on the health of both parturient women and their children [1]. If one takes into consideration the institutionalization of the act of giving birth, then, it is possible understanding the belief of both health professionals and the overall population that giving birth in hospital environments makes childbirth safer [1, 2] Therefore, several interventions were developed and remain used during labor, delivery and birth [3]. Childbirth is often medicalized, a fact that limits women’s rights of choice and triggers feelings such as fear and insecurity [4].

Although intervening in labor is a common practice, this attitude goes against the current scientific evidence [3]. The adoption of clear clinical protocols and criteria, based on scientific evidence, enables one’s decision about intervening substantiated by factors rather than just by clinical experience [3]. The belief that all interventions in labor - mainly those of habitual risk - help improving maternal and neonatal outcomes has been refuted [4, 5]. Current evidence has shown that the excessive use of interventions can lead to unnecessary cesarean sections, as well as to iatrogenic prematurity and postpartum depression, among others [6–8].

Thus, since 1996, the World Health Organization (WHO) recommends interventions based on scientific evidence. This set of recommendations was named as good care practices in natural childbirth [9]. Such practices have been classified into four categories: practices that are clearly useful and should be encouraged; practices that are clearly harmful or ineffective and must be ruled out; practices inappropriately used during labor and delivery; and practices that should be investigated due to lack of scientific evidence [4]. WHO (2018) has updated the document issued in 1996, based on Sustainable Development Goals 3 (SDG) [10]. The focus of it was not only to avoid complications in childbirth, but also to make the experience of giving birth positive and to enable better physical, mental and psychological outcomes, both for the mother and the baby [10].

More than 20 years after the first WHO recommendations, the use of interventions lacking scientific evidence during labor remains a challenge in several countries [6, 11–13]. A study carried out in Spain has shown high levels of interventions during labor. Kristeller maneuver stood out among them, although scientific evidence has already shown the risks posed by this maneuver both to the mother and the baby [11]. Two out of three pregnant women in Turkey were subjected to elective labor induction, and this procedure contradicts the current evidence that discourages this practice [6]. The rate of interventions in childbirth in Australia has increased by 5 and 15 % in public and private hospitals, respectively, in recent years. In addition, only 15 out of every 100 women in private hospitals were not subjected to interventions in childbirth [12]. A study carried out in the Philippines has corroborated these results and highlighted a gap between recommendations based on scientific evidence and clinical practices [14].

More than 20 years after the first proposal, some interventions that should have been ruled out remain in place in Brazil [13]. On the other hand, clearly useful practices still face barriers for their implementation during parturition processes [12].

A study conducted in Brazil has evaluated the use of good practices, such as eating, walking, and adopting non-pharmacological methods for pain relief during labor of usual-risk pregnant women with physiological pregnancy, without morbidities [15]. Results have shown that these practices were available for less than 50 % of women [13]. On the other hand, interventions such as venous catheter, lithotomy position at delivery time and oxytocin using recorded high prevalence in this population [13]. The aforementioned study has also shown the magnitude of regional inequalities in the use of good care practices in childbirth, since Northern, Northeastern and Midwestern Brazil recorded the lowest prevalence of clearly useful and beneficial practices, as recommended by WHO, in 1996 [13]. These results have highlighted inequalities in health, since practices that should be implemented during childbirth recorded lower prevalence in these regions [16]. On the other hand, these very same practices were more prevalent in Southeastern Brazil, the most economically developed region in the country [13, 16, 17].

The fact that some interventions in childbirth lead to the use of other interventions and contribute for pregnant women’s body to be subjected to several interventions (that are not associated with improved neonatal outcomes) is another factor that deserves attention [12]. The coexistence of these interventions in the same labor depicts a technocratic care model that reduces and limits women’s empowerment in their own childbirth [18]. In addition, the imposition of these interventions often violates women’s rights, leads to iatrogenic actions and dehumanizes the obstetric practices [19].

Several studies about obstetric interventions were carried out in the past decades [13, 14, 16–18]. However, such interventions were often evaluated in separate [13]. Thus, assessing the coexistence of obstetric interventions is particularly important to protect women’s and collective health, since it helps better understanding the likely factors (sociodemographic, obstetric, and hospital obstetric model) influencing the whole series of interventions women are subjected to. Moreover, it can provide evidence to solve the gap in knowledge about the coexistence of obstetric interventions.

The aims of the current study were to assess the prevalence and coexistence of obstetric interventions performed in maternity hospitals in Belo Horizonte City (MG), based on the Grade of Membership (GoM) method, as well as to investigate sociodemographic and obstetric factors associated with the coexistence of profiles generated by the aforementioned method.

## Methods

Observational study, based on cross-sectional design, conducted with data deriving from the study “Born in Belo Horizonte: Survey on Childbirth and Birth”, in 7 maternity hospitals belonging to the public health network and in four maternity hospitals that assist the supplementary health network of Belo Horizonte City - Minas Gerais State, Brazil.

The survey “Born in Belo Horizonte - Survey on childbirth and birth” adopted the same sampling method, logistics, and material resources followed by the nationwide study entitled “Born in Brazil - Survey on childbirth and birth” - details can be checked in a previously published study [20].

The current study included all women in labor (L), either induced or spontaneous, regardless of the delivery route, who were admitted to the investigated maternity hospitals − 1,088 women, in total. The herein conducted analysis excluded women who delivered by cesarean section or who did not have information about obstetric interventions - the final sample comprised 588 parturient women.

Data were collected by trained nurses in two data sources, from November 2011 to March 2013. Parturient women’s medical records were the first data source, whereas the second one was featured by one-on-one interviews conducted at least 6 h after delivery. This time was pre-established as the minimum interval for postpartum rest. The remaining data were directly collected from patients’ medical records. Time difference between data collection years and the analysis of this proposal did not compromise the current results because, assumingly, there was no temporal dissociation in variables and in the study design.

Data collection was based on two different sources, namely: standardized questionnaire applied to the mothers in order to collect identification data, as well as sociodemographic and clinical history variables, among others; and the medical records of parturient women. Variables such as providing food to parturient women, allowing them to have freedom to move, adopting non-pharmacological methods (NPM) for pain relief, “lying parturient women on their back with legs up” at delivery time, analgesia and episiotomy were collected from parturient women’s’ medical record and during the interview.

The research team has made the option for using data collected during interviews, because its members believed that puerperal women’s perception was more reliable. Variables such as amniotomy, oxytocin infusion, perineal shaving, enema and use of partogram were collected from patients’ medical records, since they were the ones most often recorded in it.

Variables selected to build the obstetric intervention coexistence profiles were based on the classification of obstetric practices put in place during labor and delivery that were suggested by the World Health Organization (WHO), since this classification was adopted at data collection time, as shown in (Table 1).

**Table 1:** Description of study variables

Obstetric practices put in place during labor and delivery		
Variables	Collection instrument	Categories
*Useful practices that should be encouraged*		
Providing food to parturient women	Interview	Yes; no; non-informed
Allowing parturient women to have freedom to move	Interview	Yes; no; non-informed
Use of Partogram (tool used to assess labor evolution)	Medical Record	Yes; no
Adopting Non-Pharmacological Methods (NPM) for pain relief	Interview	Yes; no; non-informed
*Harmful or ineffective practices that must be eliminated*		
Enema	Medical Record	Yes; no
Perineal shaving	Medical Record	Yes; no
“Lying parturient women on their back with legs up” - position at delivery time	Interview	Yes; no; non-informed
Kristeller maneuver	Interview and medical record	Yes; no; non-informed
*Practices inappropriately used at L and delivery time*		
Amniotomy	Medical Record	Yes; no; non-informed
Oxytocin infusion	Medical Record	Yes; no; non-informed
Analgesia	Medical Record	Yes; no
Episiotomy	Medical Record	Yes; no; non-informed

*NPM* Non-pharmacological methods for pain relief, *L* labor

The current study has used GoM to identify the coexistence of the adopted obstetric interventions. This method allows diffuse pertinence, i.e., it allows individuals to partially belong to more than one group, rather than to organize themselves in well-defined groups, as seen in other methods [21].

The aforementioned method estimates a relevance degree score for each individual in comparison to different groups. It is applied to a data set comprising *i* individuals (*i* = 1, 2, …, I), with *j* categorical variables (*j* = 1, 2, …, J). Each *j-th* variable has *Lj* response levels. Discrete response variable *Xijl* is predicted by two sets of generated coefficients: *λkjl* and *Gik*. *λkjl* refers to the likely incidence of a given attribute between pure types of the profile and it can assume any value between 0 and 1. The model estimates a pertinence degree score (*Gik*) for each individual; this score represents the degree to which element *i* belongs to the extreme profile - it ranges from 0 to 1, which corresponds to 100 % of profiles classified as extreme (*k*) [21, 22].

The preponderance criterion, which constitutes the *λkjl*/marginal frequency ratio (E/O ratio), establishes an objective criterion to feature the generated extreme profiles. Marginal frequency can be understood as the likely incidence of such a feature in the total population. The likely incidence of *l-th* response to the *j-th* variable in the *k-th* profile among pure types of the referred profile must be at least 20 % (cut-off value) higher than the observed marginal likelihood [21, 22].

The number of extreme profiles *k* was predetermined for each round of GoM. Analysis was performed based on six different models (*k* = 2, 3, 4, 5 and 6 profiles). The Akaike Information criterion (AIC) was used to define the most appropriate representation model (tested up to *k* = 6). The decision rule corresponded to the model recording the minimum AIC statistical value [21–24].

Parameters “*Gik*” and “*λkjl*” were estimated in the GoMRcpp.R software for R [19]. The prevalence of extreme profiles in the analyzed population was calculated as follows:

$$P_k=\frac{\sum_{i=l}^lg_{ik}}{\sum_{i=l}^li}\;with\;k=1,\;2,\;\dots,\;K$$

Such a prevalence can be considered a weighted average because the weight corresponds to the proportion of the population that does not show degree of relevance to the referred profile greater than 0 and lower than, or equal to, 1.

Statistical analyses in the current study were performed in Stata software, version 16.0. Estimates were presented in proportions (%), at 95 % Confidence Interval (95 % CI). Quantitative variables were subjected to Shapiro-Wilk test to check data asymmetry - asymmetric data were expressed as median and interquartile range (IQ).

After the profiles were built and the degree of belonging (G_iK_) of each woman was defined, they were separated based on the highest degree of belonging to the profile. They were categorized as belonging to profile 1 whenever their G_iK_ was ≥ 0.5 for profile 1 and as belonging to profile 2 whenever G_iK_ was < 0.5 for profile 2.

Poisson regression models were built to estimate parameters of interest – such as gross and adjusted for age, skin color, schooling, number of prenatal consultations, gestational age at delivery time, and presence of companion during labor and delivery. It was done to investigate the magnitude of association between profiles generated by the coexistence of obstetric interventions and their likely determinants (exposure variables).

The error recorded for the relative risk can be overestimated when this regression model is applied to binomial data, since the Poisson distribution variance increases progressively, whereas that of the binomial distribution presents its maximum value when prevalence reaches 0.5. Robust variance estimator was used to fix this problem, in order to find results similar to those recorded through Mantel-Haenszel statistics, which was used when the covariate of interest was categorical [25].

The multivariate regression model adopted the backward method to build the adjusted model; all variables of interest presenting statistical significance level lower than 20 % were included in the bivariate analysis. Theoretical criteria were also taken into consideration in the current study. Hosmer & Lemeshow test was used to check the fit of the final model. Crude and adjusted prevalence ratios (PR) were presented and the 95 % CI was calculated, by taking into consideration significance level of 0.05 in all analytical procedures.

## Results

Data about 588 parturient women were analyzed. Their median age was 26 years (IQ = 21–31 years) – women who self-reported brown skin color (69.56 %), who had a paid job (76.77 %), finished high school (56, 97 %) and lived in stable union regime (66.67 %) have prevailed in the sample (Table 2).

**Table 2:** Sociodemographic profile of the investigated mothers. Belo Horizonte City, Minas Gerais State, Brazil

	n(%)	IC95 %
**Age**^a^	26 (21–31)	
**Skin Color**		
White	125(21.26)	18.13–24.76
Black	54(9.18)	7.09–11.80
Brown^b^	409(69.56)	65.70–73.15
**Paid job**		
No	313(53.23)	49.17–57.24
Yes	275(76.77)	42.75–50.82
**Schooling**		
Illiterate and Elementary School	202(34.35)	30.61–38.29
High school	335(56.97)	52.92–60.93
Major degree	51 (8.67)	6.64–11.24
**Marital Status**		
With companion	392(66.67)	62.74–70.37
Without companion	196(33.33)	29.62–37.25

Notes: ^a^Median (IQ); ^b^Includes Asian-descendant and indigenous individuals; *95 % CI* Confidence Intervals

Table 3 shows the λ_kjl_ coefficients recorded for variables of obstetric interventions, which were selected based on the classification of obstetric practices put in place during labor and delivery, based on suggestions by WHO. Up to 6 profiles were generated (k = 6) - the profile presenting k = 2 recorded the lowest AIC value for all GoM analyses.

Women recording total belonging (*G*_*ik*_ = 1) to profile 2 presented the following features: they did not receive any food during labor and delivery, did not have freedom to move, did not present partograph using record, were not administered with NPM for pain relief, were subjected to enema procedure and underwent Kristeller maneuver. Moreover, they received analgesia and underwent episiotomy (Table 3).

**Table 3:** Distribution of lambdas coefficients recorded for internal variables in each extreme profile of obstetric intervention patterns observed in hospitals in Belo Horizonte City, Minas Gerais State, Brazil

Obstetric Interventions	n (%)	Profile 1 (λ1_jl_)	Profile 2 (λ2_jl_)	Profile 1(E/O ratio)	Profile 2(E/O ratio)
*Useful practices that should be encouraged*					
**Providing food to parturient women**					
Yes	185(31.463)	0.552	0.000	**1.754**	0.000
No	375(63.776)	0.448	0.888	0.703	**1.392**
Non-informed	28(4.762)	0.000	1.124	0.000	**2.360**
**Allowing parturient women to have freedom to move**					
Yes	353(60.03)	1.000	0.000	**1.666**	0.000
No	78(13.265)	0.000	0.324	0.000	**2.443**
Non-informed	157(26.70)	0.000	0.676	0.000	**2.532**
**Use of Partogram**					
Yes	377(64.12)	0.795	0.432	**1.239**	0.674
o	211(35.88)	0.206	0.568	0.573	**1.583**
**Adopting Non-Pharmacological Methods (NPM) for pain relief**					
Yes	329(55.952)	1.000	0.000	**1.787**	0.000
No	234(39.796)	0.000	0.900	0.000	**2.261**
Non-informed	25(4.252)	0.000	0.100	0.000	**2.354**
*Harmful or ineffective practices that must be eliminated*					
**Enema**					
No	583(99.150)	1.000	0.980	1.009	0.988
Yes	5(0.50)	0.000	0.020	0.000	**2.352**
**Perineal shaving**					
No	583(99.150)	1.000	0.980	1.009	0.988
Yes	5(0.850)	0.000	0.020	0.000	**2.352**
“**Lying parturient women on their back with legs up” - position during delivery**					
No	474(80.612)	0.753	0.876	0.936	1.087
Yes	83(14.116)	0.247	0.000	**1.747**	0.000
Non-informed	31(5.272)	0.000	**0.124**	0.000	**2.354**
**Kristeller maneuver**					
No	357(60.714)	1.000	0.000	**1.647**	0.000
Yes	196(33.33)	0.000	0.857	0.000	**2.572**
Non-informed	35(5.952)	0.000	0.143	0.000	**2.397**
*Practices inappropriately used at labor and delivery time*					
**Amniotomy**					
No	255(43.367)	0.524	0.310	**1.207**	0.715
Yes	121(20.578)	0.185	0.235	0.898	1.141
Non-informed	212(36.054)	0.292	0.445	0.809	**1.263**
**Oxytocin infusion**					
No	298(50.680)	0.579	0.401	1.143	0.809
Yes	290(49.320)	0.421	0.590	0.853	1.197
**Analgesia**					
No	422(71.769)	1.000	0.338	**1.393**	0.471
Yes	166(28.23)1	0.000	0.662	0.00	**2.346**
**Episiotomy**					
No	420(71.429)	**1.200**	0.351	**1.400**	0.491
Yes	152(25.850)	0.000	0.585	0.000	**2.264**
Non-informed	16(2.721)	0.000	0.064	0.000	**2.356**

Notes: AIC values based on models with two, three, four, five and six profiles were: 10,114; 10,412; 11,000; 11,000 and 12,0000 / λ1jl = lambdas

Table 4 shows the weighted prevalence and the prevalence of *G*_*ik*_ = 1 for each generated profile. Profile 1 recorded the highest weighted prevalence (57.59 %) of belonging and it can be considered the profile in which good care practices in childbirth and birth were prevalently used. Profile 2 recorded high weighted prevalence (42.24 %) of belonging; it was featured as the profile in which the largest number of good practices were not used, based on the classification by WHO. However, this profile presented the largest number of obstetric interventions, i.e., practices that are harmful or ineffective and that must be ruled out, or, yet practices that are inappropriately used at labor and delivery time. There was also prevalence of *G*_*ik*_ = 1 in this profile (in 11.05 % of the sample), in other words, these women fully belonged (100 %) to this profile (Table 4).

**Table 4:** Prevalence of each extreme profile of obstetric intervention patterns observed in Hospitals in Belo Horizonte City, Minas Gerais State

Profiles	Weighted prevalence	*G*_*ik*_ = 1
**Profiles 1**		**n (%)**
Parturient women who were offered food, parturient women allowed to have freedom to move, use of partogram, adoption of non-pharmacological methods for pain relief, giving birth in lying position, lack of Kristeller maneuver and amniotomy, no oxytocin infusion and analgesia using, and lack of episiotomy	57.59	142 (24.15 %)
**Profiles 2**		
Parturient women who were not offered food and did not have freedom to move, who did not use partogram and non-pharmacological methods for pain relief, as well as who underwent enema, Perineal shaving, Kristeller maneuver, amniotomy, oxytocin infusion, analgesia and episiotomy	42.24	65 (11.05 %)

Table 5 shows bivariate analyses of likely factors associated with the generated profiles. There was association between profiles and variables such as age, schooling, skin color, hospital financing, as well as presence and performance of obstetric nurse at the institution.

**Table 5:** Bivariate analysis of factors associated with profiles in hospitals. Belo Horizonte City, Minas Gerais State

Variable	Profile 1	Profile 2	RR (CI 95 %)
*Sociodemographic variables*			
** Age (years)**	25.72(5.88)	26.87(6.88)	1.01**(1.00–1.03)**
**Schooling**			
Illiterate and Elementary school	128(37.43)	74(30.08)	1
High school	197(57.60)	138(56.10)	1.12(0.90–1.40)
Major degree	17(4.07)	34(13.82)	1.81(**1.39–2.37**)
**Skin color**			
White	61(17.84)	64(26.02)	1
Black	33(9.65)	21(8.54)	0.75(0.52–1.10)
Asian-descendant	248(72.51)	161(65.45)	0.76(**0.63–0.94**)
**Paid job**			
No	190(55.56)	123(50.00)	1
Yes	152(44.44)	123(50.00)	1.13(0.94–1.37)
*Obstetric variables*			
**Number of prenatal consultations**	7.80(2.69)	7.92(2.88)	1.00(0.97–1.04)
**Primigravida**			
Yes	147 (42.98)	117(47.56)	1
No	195(57.02)	129(52.44)	0.89 (0.74–1.08)
**Gestational age at delivery (weeks)**	38.72(2.09)	38.65(2.08)	0.99 (0.94–1.03)
**Financing the delivery institution**			
Public	338(98.83)	180(73.17)	1
Private	4(1.17)	66(26.83)	2.71**(2.37–3.09)**
**Childbirth Hospital with Obstetric Nurse**			
Absent	87(25.44)	142(58.68)	1
Present	51(14.91)	62(25.62)	0.88(0.72–1.07)
Active	204(59.65)	38(15.70)	0.25**(0.18–0.34)**
**Companion during childbirth**			
Yes	331(96.78)	237(96.34)	1
No	11(3.22)	9(3.66)	1.07(0.65–1.76)

Notes: Value in bold: *p* <0.05; *RR* Relative risk; *95% CI* 95% confidence interval

Table 6 shows the final Poisson regression model adjusted for the following variables: age, skin color, schooling, number of prenatal consultations, gestational age at delivery, and presence of companion during labor and delivery.

The presence of acting obstetric nurse in the hospital where women gave birth has reduced by 0.35 times, on average, the prevalence of belonging to the profile in which good practices were not prevalent, in comparison to women who gave birth in hospitals where this professional was not available.

With respect to variable “hospital financing type”, women who gave birth in private hospitals have shown increase by 1.99 times, on average, in the prevalence of belonging to profile 2 in comparison to women who gave birth in public hospitals.

**Table 6:** Final Poisson regression model. Belo Horizonte City, Minas Gerais State

Variables	PR	CI 95 %	*p*-value
**Hospital with availability of obstetric nurse**			
Absent	1		
Present	1.11	0.85–1.46	0.412
Active	0.35	**0.25–0.49**	**< 0.001**
**Hospital financing type**			
Public	1		
Private	1.99	**1.60–2.49**	**< 0.001**

Notes: *PR* prevalence ratio; *95 % CI* 95 % confidence intervals; Bold value = *p* < 0.05

## Discussion

The aim of the current study was to assess the prevalence of obstetric interventions and profiles generated by the coexistence of obstetric interventions, as well as sociodemographic and obstetric factors associated with these profiles. Results have shown two antagonistic obstetric profiles. Profile 1 generated in the current study comprised parturient women who were offered food, had freedom to move, were subjected to use of partogram and to non-pharmacological methods for pain relief, gave birth in lying position, did not undergo Kristeller maneuver and amniotomy, as well as who were not subjected to oxytocin infusion, analgesia and episiotomy. Profile 2 comprised parturient women who were not offered food, were not free to move, were not subjected to use partogram or non-pharmacological methods for pain relief, and who were subjected to enema, perineal shaving, Kristeller maneuver, amniotomy, oxytocin infusion, analgesia and episiotomy. Based on the analysis of factors that have influenced the coexistence of obstetric interventions, the presence of obstetric nurses in the healthcare practice has reduced the likelihood of parturient women to join profile 2. In addition, childbirth events that took place in public institutions have reduced the likelihood of parturient women to join profile 2.

It is necessary understanding the benefits and risks of each intervention, in separate, and, later on, the influence of their coexistence on health outcomes observed in mothers and newborns must be investigated in order to better understand differences between generated profiles. Practices, such as freedom to move, use of partogram, adoption of non-pharmacological methods for pain relief, among others, are not limited to clinical conditions; they can be used to assist labor, as long as parturient women present stable clinical conditions [10].

With regards to clearly useful practices that should be encouraged, one can say that they provide a safe and respectful environment for physiologic labor to take place. Accordingly, labor development should be monitored with the aid of partogram. Partogram has been incorporated to several services [26]. Its use allows monitoring labor based on graphical analysis, as well as defining whether, or not, the professional controlling the whole process needs to intervene in it [26]. Another practice that should be adopted is to encourage women to eat during labor to ensure the energy they need to experience the whole process of giving birth [27]. In addition, freedom of movement and labor verticalization are encouraged practices because they help the physiological process of childbirth and allow pain relief [28]. Above all, parturient women are encouraged to take the most comfortable position in labor [29, 30].

The use of non-pharmacological methods comprising several techniques - such as massage, hydrotherapy and aromatherapy - applied during labor and delivery to relieve and control pain is another encouraged practice [28, 31]. These techniques are encouraged because they bring comfort and well-being to parturient women, as well as contribute to bond between the mother and the professional [31].

Enema and perineal shaving also form this group; there is no evidence to support their use, which is uncomfortable to women [32–34]. This maneuver is still performed in Brazilian hospitals, despite the clear evidence that it should be ruled out [32].

Amniotomy, oxytocin infusion, analgesia and episiotomy form the group of inappropriately used practices [9, 10]. Amniotomy is often used to speed up parturition processes by increasing uterine contractions, even without any clinical indications [35]. This practice is selectively encouraged, due to the risks and benefits provided by it, and it must be based on clear clinical protocols and criteria [36]. In addition to increase maternal discomfort, it poses the risk of umbilical cord contamination and prolapse [35]. Synthetic oxytocin belongs to the list of drugs with high damaging power, based on the American Institute for Safe Medication Practices [37]. Oxytocin using should be evaluated based on clear clinical criteria substantiated by scientific evidence, since it can increase maternal discomfort, as well as the risk of uterine hypertonia and placental detachment [36, 37]. Elective induction, or its use only to accelerate labor without clinical indications based on scientific evidence, should be discouraged [36, 37]. Analgesia in vaginal delivery should be guaranteed and made available for parturient women, if they ask for it [38]. However, it is worth emphasizing that this procedure should always be weighed due to likely risks for both the parturient women and the baby [10, 38]. The use of other non-pharmacological methods for pain relief is recommended [39]. Current evidence points towards the harm caused by episiotomy; there is no evidence that it reduces the number of negative perinatal outcomes [40, 41].

The existence of two antagonistic profiles comprising women with similar obstetric profiles has evidenced the coexistence of two provided-assistance models. On the one hand, there is the model adopting useful technologies to help providing women undergoing parturition processes with comfort. Therefore, it meets the assumptions of assistance based on scientific evidence, as recommended by WHO, and reduces the risks posed by unnecessary interventions [10]. On the other hand, there is the assistance model that is not based on scientific evidence, given the use of clearly harmful practices that must be ruled out [42]. The use of interventions lacking scientific evidence increases the risk of iatrogenic behaviors that, in addition to put mother and baby at risk, contribute to the development of fear and postpartum depression [43]. The technocratic model persists, despite all the evidence supporting changes in care practices [44]. This finding corroborates other studies, according to which, it is necessary implementing continuing education actions, as well as audits to support and guide the clinical practice, in order to change the healthcare model [3, 45].

Based on the analysis of factors associated with the generated profiles, hospitals that had an active obstetric nurse reduced by 0.35 times, on average, the prevalence of belonging to profile 2. This result corroborates previous research, which has shown that intervention rates decrease whenever obstetric nurses provide childbirth care for women at habitual risk [46]. Another evidence has shown that there are better maternal and neonatal outcomes whenever childbirth assistance, based on good practices, is provided by obstetric nurses working in the delivery scenario [4, 47–49].

Decree n. 94,406, from June 8, 1987, which regulated law n. 7,498, from June 25, 1986, which, in its turn, addressed the exercise of Nursing in Brazil, guaranteed the performance of obstetric nurses in childbirth assistance [50]. The National Health Agency (ANS - Agência Nacional de Saúde) has published Normative Resolution n. 398 in 2016, which required health insurance companies to hire obstetric nurses, whenever feasible [51]. This normative resolution represents an advancement for women’s health, although inspection difficulties are an obstacle to its implementation [52]. Hiring obstetric nurses does not guarantee their effective performance in the delivery scenario, since these professionals are often limited to bureaucratic processes [52, 53]. Therefore, in addition to guarantee their hiring, it is necessary allowing these professionals to act directly in childbirth assistance [52].

With respect to hospital financing, women who gave birth in private hospitals were more likely to belong to profile 2 than women who gave birth in public hospitals. A nationwide study has shown significant increase in the access to some technologies during childbirth, as well as reduced number of harmful practices in the private sector, from 2011 to 2017. This outcome has evidenced slight advancement in the private sector [4]. However, the prevalence of practices that are clearly harmful or ineffective and that must be ruled out, or of practices that are inappropriately used nowadays, remains higher in the private sector [4].

The relevance of public institutions in assisting women in Brazil is widely acknowledged, since they help reducing the number of unnecessary cesarean Sec [4]. Women assisted in public hospitals have more access to good care practices at childbirth and birth than those assisted in private institutions [4, 54]. This fact may be associated with actions and incentives carried out by the Brazilian Ministry of Health (MS - Ministério da Saúde), based on recommendations by WHO, to promote humanized and vaginal delivery. The Brazilian Ministry of Health has encouraged institutions to adopt good care practices in childbirth by elaborating handbooks, issuing ordinances, developing environment adaptations and by qualifying professionals working in the delivery and birth scenarion [55].

Finally, it is necessary highlighting some limitations of the current study, such as the fact that the sample is not representative of Belo Horizonte City and that some data were collected through interviews carried out after delivery, which may have affected the report by some women. However, it is worth emphasizing that the time difference between data collection years and the analyses of the current proposal did not compromise the current results since, assumingly, there was no temporal dissociation in variables (temporal ratio of that time was analyzed), in study period design and in the context inherent to the combined influence of obstetric interventions in the Brazilian obstetric scenario.

The current study represents an advancement in its research field, since it has analyzed the coexistence of obstetric interventions and the features influencing their emergence. It is noteworthy that most studies often investigate the use of obstetric interventions in separate, without taking into account that interventions coexist and influence one another, a fact that potentiates positive or negative outcomes at childbirth and birth.

## Conclusions

The current study has evidenced that obstetric interventions were carried out in combination. Two antagonistic profiles of obstetric intervention coexistence were generated for women presenting similar obstetric profiles: the first profile was featured by clearly useful obstetric interventions that benefit parturient women, whereas the second profile took into account clearly harmful practices that must be ruled out.

It has also evidenced factors associated with the coexistence of the generated profiles and has contributed to discussions about childbirth assistance in Brazil. In addition, it has emphasized the need of building strategies focused on promoting adherence to, and the implementation of, assistance models based on scientific evidence.

The current study has contributed to discussions about obstetric interventions and childbirth assistance models aimed at implementing public policies to ensure assistance based on good practices at childbirth and birth.

## Data Availability

Datasets used and/or analyzed in the current study will be made available by the corresponding author upon reasonable request.
